# Evaluation of low and high interstitial glucose concentrations in healthy, nondiabetic dogs using a flash glucose monitoring system

**DOI:** 10.1093/jvimsj/aalag032

**Published:** 2026-03-06

**Authors:** Rebecca F Brisman, Douglas A Palma, Philip R Fox

**Affiliations:** The Schwarzman Animal Medical Center, New York, NY 10065, United States; The Schwarzman Animal Medical Center, New York, NY 10065, United States; The Schwarzman Animal Medical Center, New York, NY 10065, United States

**Keywords:** canine, diabetes mellitus, freestyle libre, interstitial glucose

## Abstract

**Background:**

Flash glucose monitoring systems (FGMS; FreeStyle Libre) are useful devices for managing diabetic patients. The FGMS is reportedly accurate for diabetic dogs with hyperglycemia and euglycemia but might underestimate glucose concentrations during hypoglycemia.

**Hypothesis/Objectives:**

Assess the frequency of low and high interstitial glucose (IG) concentrations recorded in healthy, nondiabetic dogs using FGMS.

**Animals:**

Twenty-three hospital employee-owned dogs.

**Methods:**

Prospective, observational study. The FGMS was placed on all dogs to record ≥488 readings each over up to 14 days. Interstitial glucose concentrations were analyzed to identify the frequency of low, normal, and high IG concentrations. Descriptive statistics were calculated, and comparisons between demographic cohorts were performed.

**Results:**

During monitoring, 73.7% (14/19) of participants had at least one low IG concentration (<70 mg/dL), whereas 26.3% (5/19) had at least one high IG concentration (>180 mg/dL). The mean (±SD) percentage of low and high IG concentrations per dog was 2.8 ± 4.3% and 0.8 ± 2.2%, respectively*.* Markedly decreased IG (<55 mg/dL) and markedly increased (>250 mg/dL) IG concentrations occurred in 63.2% (12/19) and 10.5% (2/19) of dogs, respectively. The frequency of low IG concentrations in dogs weighing 2.5-20.5 kg (2.2%; interquartile range [IQR], 1.1-5.0) was higher (*P* = .02) than in dogs weighing 20.6-41.4 kg (0.1%; IQR, 0.0-0.7%); the median difference was 2.1% (95% confidence interval, 0.6-10.1).

**Conclusions and clinical importance:**

Low and high IG concentrations were recorded in healthy, nondiabetic dogs, providing a context for interpreting FGMS results in diabetic dogs.

## Introduction

Regular glucose monitoring is a vital component of successful management of diabetes mellitus. Traditionally, veterinarians have relied on blood glucose curves to assess glycemic control and insulin response. Using this method, pets typically are hospitalized for 10-12 hours with capillary blood glucose concentration measured every 2 hours. Some pet owners can perform these curves at home using portable blood glucose meters. However, both approaches are labor-intensive, require multiple needle punctures, provide only intermittent data, and might miss day-to-day variability in blood glucose concentrations.^1–4^

In recent years, continuous glucose monitoring systems (CGMS), including the flash glucose monitoring system (FGMS), have addressed many of these limitations. Compared with hospital-based curves, CGMS allows animals to remain at home and maintain normal routines, decreasing stress and yielding more representative glucose profiles. Additionally, CGMS provide nearly continuous data, minimizing the risk of missing important fluctuations in blood glucose concentrations.^1–6^

Among the available FGMS devices, the 14-day FreeStyle Libre (Abbott) reports interstitial glucose (IG) concentrations from 40 to 500 mg/dL.^7,8^ This FGMS has been validated in diabetic dogs without comorbidities, demonstrating accuracy during hyperglycemia and euglycemia. However, its reliability decreases in diabetic dogs experiencing hypoglycemia (blood glucose concentrations <70 mg/dL), with a low correlation (*r* = 0.43) between blood glucose and IG concentrations.^1^ In healthy nondiabetic dogs, it failed to reliably detect hypoglycemia with rapidly changing blood glucose concentrations. This discrepancy might in part reflect the physiologic lag between changes in blood and IG concentrations because glucose must diffuse from the intravascular to the interstitial space before being detected by the sensor.^2^ Although studies in people suggest the FreeStyle Libre 2 offers improved accuracy in the hypoglycemic range compared to its predecessor, a FreeStyle Libre 2 accuracy study conducted in healthy, nondiabetic dogs found that only 50.9% of low-range readings in healthy dogs fell within 15 mg/dL of a reference standard during experimentally-induced hypoglycemia.^9,10^

Pet owners find FGMS more user-friendly and effective for diabetes mellitus management than traditional methods, citing improved control and ease of use. However, continuous access to their pets’ glucose concentrations via FGMS can lead to increased anxiety, especially regarding hypoglycemia, which may negatively impact owners’ quality of life.^11,12^ It has not been established whether at-home glucose monitoring decreases hypoglycemia-related anxiety in pet owners.^12^

Given the increasing role of FGMS in diabetes management, understanding FGMS baseline glucose patterns in healthy, nondiabetic dogs is important. Such data could inform interpretation of low IG and high IG concentrations in diabetic dogs and may help alleviate owner anxiety associated with CGMS. We aimed to (1) evaluate the frequency of low IG concentrations in healthy dogs using FGMS and (2) assess the frequency of high IG concentrations in the same population. Given the reported limitations of FGMS in accurately detecting hypoglycemia, we hypothesized that normal, nondiabetic dogs would commonly exhibit low IG concentrations (<70 mg/dL) when monitored with FGMS.^1,2^ We sought to quantify the frequency of such events in healthy, non-diabetic dogs and to establish baseline data to inform interpretation of FGMS results in diabetic dogs.

## Materials and methods

### Animals

Healthy hospital employee-owned dogs were prospectively enrolled in an observational study between December 2024 and February 2025. To be included, dogs had to be at least 6 months old and have a body condition score (BCS) ≥4/9 and ≤6/9.^13^ Exclusion criteria included concurrent disease, use of medications that can cause hyperglycemia or hypoglycemia (eg, insulin, corticosteroids, some antibiotics, calcineurin inhibitors, thiazides, angiotensin converting enzyme inhibitors, beta blockers), and use of medications that can impact FGMS results (eg, acetaminophen, dopamine, icodextrin, salicylates, ascorbic acid). Participants could not undergo anesthesia, radiography, computed tomography, diathermy treatment, or magnetic resonance imaging while the device was in place.^8,14,15^ Owners completed a survey to determine eligibility, in which all patient medications and comorbidities were described (https://forms.gle/XVxEJ4vj3pgYkfrt7). To screen for clinically relevant abnormalities that could influence insulin resistance, all enrolled participants had a CBC and serum biochemistry panel performed less than 2 weeks before initial device placement. The study was approved by the Institutional Animal Care and Use Committee at the Schwarzman Animal Medical Center. All dogs were privately owned, and informed consent was obtained from owners before enrollment.

### Data collection

A FreeStyle Libre 2 FGMS sensor was placed on each dog as previously described, either by the primary author or by an internal medicine service technician.^1^ Briefly, skin at the area of the dorsal neck was clipped and cleaned with isopropyl alcohol. Four drops of tissue adhesive (GLUture, Zoetis Inc.) were placed on the adhesive surface of the sensor, and then the sensor was placed according to the manufacturer's guidelines. The sensor was allowed 1 hour to acclimate before any readings were obtained. Each dog had its own FGMS sensor. Owners were instructed to feed, exercise, and give treats to their pets normally while the device was in place. A single device could obtain readings for up to 14 days. If a device became detached or malfunctioned and had to be replaced, then cumulative data were collected and analyzed which could be more than 14 days. If the device was still attached after 14 days, it was removed by either the owner or a trained veterinary professional. Glucose data were uploaded automatically to the FGMS online platform (LibreView, Abbott) using each owner’s FreeStyle Libre account. The investigator’s LibreView account was linked to each pet owner’s account to allow secure remote access, review, and to download IG data for analysis.

For dogs that had episodes of markedly increased IG concentrations (>250 mg/dL), a serum biochemistry panel, urinalysis, and serum fructosamine concentration were measured within 10 days of the event. Owners of these participants also were contacted to provide information about clinical signs and activity at the time of the high IG concentrations. Because low IG concentrations were anticipated based on the study hypothesis, additional follow-up testing was not performed for those participants. However, all owners were instructed to report any adverse events or concerns observed while their dogs were wearing the FGMS, and no such reports were received.

### Flash glucose monitoring system

The FGMS, FreeStyle Libre 2, is composed of a 35 × 5 mm round sensor with a thin 0.4 × 5 mm catheter that is inserted under the skin and measures glucose concentrations in the interstitial fluid. The sensor, based on the glucose oxidase method, is factory-calibrated and eliminates the need for daily blood sampling. Using the applicator provided by the manufacturer, the sensor is applied directly on the skin and can be worn for up to 14 days. The sensor measures IG concentrations every minute and stores an average of these concentrations every 15 minutes. Data can be stored in the sensor for up to 8 hours and can be scanned by a reader or smartphone. These data can either be uploaded manually or automatically to the FGMS online platform, viewed by both clinician and owner using simplified graphics, or downloaded to Excel spreadsheets that include each data point recorded by the sensor.^7,8^

### FGMS data analysis

Online data were utilized to analyze IG concentration fluctuations. Each individual’s samples were analyzed separately, and IG concentrations were categorized according to the FreeStyle Libre manufacturer-defined cutoffs. Samples were divided into classifications of markedly decreased IG (<55 mg/dL), mildly decreased IG (55-69 mg/dL), normal IG (70-180 mg/dL), mildly increased IG (181-250 mg/dL), and markedly increased IG (>250 mg/dL). These ranges are based on reference intervals for humans, but their relevance to dogs has not been established.

### Sample size calculation

The sample size was determined with the goal of obtaining sufficient precision when estimating the frequency of IG concentrations across a wide range of concentrations. In a prior evaluation of the FreeStyle Libre system, it was reported that only 39.1% of readings in the low IG concentration range (<100 mg/dL) were within ±15 mg/dL of the reference analyzer, below the ≥95% accuracy requirement outlined in the International Organization for Standardization 15197:2013 for human-use glucometers.^2,16^ Although this figure reflects device performance during insulin-induced, rapidly changing hypoglycemia, and therefore does not represent the expected frequency of low IG concentrations in healthy dogs, it was used as a conservative benchmark for the minimum number of observations needed to estimate proportions with reasonable precision. This value did not serve as an assumption regarding the expected frequency of low IG concentrations in the study population. Using Wald’s formula for margin of error, the total number of required glucose concentrations was calculated to be 366. To account for a 25% sensor failure rate, the required number of readings was adjusted by dividing by 0.75, resulting in a final requirement of 488 readings. Each dog was expected to provide approximately 1344 readings over 14 days (one reading every 15 minutes), indicating that a single dog met the minimum reading requirement.

A power analysis was conducted to estimate the number of dogs required to characterize the mean IG concentrations across individuals, assuming a SD of 10 mg/dL, a precision of ±5 mg/dL, and 80% power. Normality was evaluated using the Shapiro-Wilk test. The Wilcoxon signed-rank test was employed because data were non-normally distributed and contained multiple zero and tied values. In such cases, exact *P*-values were not defined, and the normal approximation was applied to calculate the test statistic and *P*-values. This analysis determined that 16 dogs were required to ensure adequate data collection across multiple subjects. An inter-dog variability buffer was not included. Because each dog contributed multiple glucose concentrations, some within-subject correlation was expected. This dependence was not modeled directly in the power calculation, but the large number of readings and use of non-parametric methods helped minimize bias. Future work could use mixed-effects models to adjust for this correlation.

### Statistical analysis

For each participant, the total number and percentage of IG concentrations falling within the predefined glycemic categories were calculated. The proportion of dogs that experienced at least one event in each category was determined by identifying if a single IG concentration fell into that range. The mean ± SD percentage of low and high IG concentrations per individual also was calculated.

To summarize IG concentrations across dogs, all FGMS readings from dogs included in IG analysis were pooled, and the 2.5th and 97.5th percentiles of the distribution were calculated as descriptive lower and upper limits. Because the number of dogs and the use of repeated measurements did not meet the guidelines for establishing formal reference intervals, these limits are reported as descriptive summaries rather than true reference limits.^17^ For each dog, the median IG concentration was calculated, and potential outlier individuals were evaluated using Tukey’s method; no outliers were identified. Ninety percent confidence intervals (CIs) for the percentile limits were generated using 5000 bootstrap resamples. The minimum, maximum, mean, median, and interquartile range (IQR) of IG concentrations also were calculated to further describe the distribution.

For demographic cohort comparisons, between-group differences are reported with 95% CIs. To evaluate whether body weight was associated with the frequency of low IG concentrations, dogs were divided into a small dog group (≤20.5 kg) and a large dog group (>20.5 kg). The 20.5 kg cutoff corresponded to the median body weight of the subset of dogs included in the final data analysis. The percentage of IG concentrations in the low IG range (<70 mg/dL) was compared between groups. Because the data were not normally distributed (Shapiro-Wilk test, *P* < .05 for both groups), a Mann-Whitney U test was used.

To assess the influence of age, dogs were grouped by the median age of the cohort (≤5 years vs >5 years). The percentage of low IG concentrations was compared between groups using the Mann-Whitney U test. Additional comparisons were performed using Student’s *t* test and Welch’s *t* test to confirm that findings were consistent across parametric and nonparametric methods. Equal variance was assessed using the Brown-Forsythe test before applying parametric tests.

To investigate whether sex influenced the frequency of low IG concentrations, dogs were grouped by reproductive status (male intact, male neutered, and female spayed; no intact females were enrolled) and by sex (male and female). Comparisons across reproductive status groups were performed using the Kruskal-Wallis test, and between sexes using the Mann-Whitney U test.

All descriptive statistics and data processing were performed using Microsoft Excel (Microsoft Corporation). Inferential statistics were conducted using Python (version 3.10; Python Software Foundation) with the NumPy and SciPy libraries. All tests were 2-tailed unless otherwise specified. Statistical significance was defined as *P* < .05.

## Results

### Clinical findings

Four dogs were excluded from IG data analysis because of insufficient readings. Twenty-three individual dogs were enrolled. Eleven were spayed females and 12 were males (11 neutered, 1 intact). Breeds included 10 mixed breed dogs, 2 Yorkshire Terriers, 2 Goldendoodles, and 1 each of the following: Cavalier King Charles Spaniel, American Pit Bull Terrier, Beagle, Border Collie, Whippet, Doberman, Golden Retriever, Labrador Retriever, and Standard Poodle. The median age at the time of enrollment was 5 years (range, 7 months-15 years) and the median weight was 23.8 kg (range, 2.52-41.4 kg). The median BCS was 5 (range, 4-6).

Clinical pathology tests preceding FGMS placement recorded the following abnormalities defined by IDEXX reference ranges: lymphocytosis (*n* = 2), hypertriglyceridemia (*n* = 3), hypercholesterolemia (*n* = 3), hypoalbuminemia (*n* = 1), hyperalbuminemia (*n* = 1), decreased hematocrit (*n* = 1), hypocalcemia (*n* = 1), hypochloremia (*n* = 1), increased aspartate aminotransferase (AST) activity (*n* = 1), increased hematocrit (*n* = 1), reticulocytosis (*n* = 1), hyperglycemia (*n* = 2), hypoglycemia (*n* = 1), eosinophilia (*n* = 1), hyperphosphatemia (*n* = 1), lymphopenia (*n* = 1), increased alkaline phosphatase (ALP) activity (*n* = 1), and neutropenia (*n* = 1). These abnormalities were all mild to moderate and deemed acceptable for enrollment and unlikely to contribute to insulin resistance. Participants with hyperglycemia had serum fructosamine concentrations measured, which were within reference limits. The participant with the increased ALP activity (ALP, 679 U/L; reference range, 5-160 U/L) was evaluated for hypercortisolism, and results of the low-dose dexamethasone suppression testing and urinary cortisol-to-creatinine ratio were not consistent with hypercortisolism. The dog was not exhibiting clinical signs of hypercortisolism at the time of the study.

Throughout the study period, dogs were treated with a variety of medications including trazodone (*n* = 3; Desyrel, Teva Pharmaceuticals), amantadine (*n* = 1; Symmetrel, Endo Pharmaceuticals Inc.), fluoxetine (*n* = 3; Prozac, Eli Lilly and Company), cetirizine (*n* = 1; Zyrtec, Pfizer Labs), oclacitinib (*n* = 1; Apoquel, Zoetis Inc.), joint supplements (*n* = 1), a cannabidiol supplement (*n* = 1), and a probiotic (*n* = 2). Breed, laboratory abnormalities, and medication information are presented in Table S1.

### Evaluation of recorded IG concentrations

Online data were available for all 23 enrolled dogs. Nineteen of these dogs had at least 488 readings and were included in IG data analysis. The remaining 4 dogs were excluded from statistical evaluation because of insufficient data.

**Figure 1:**
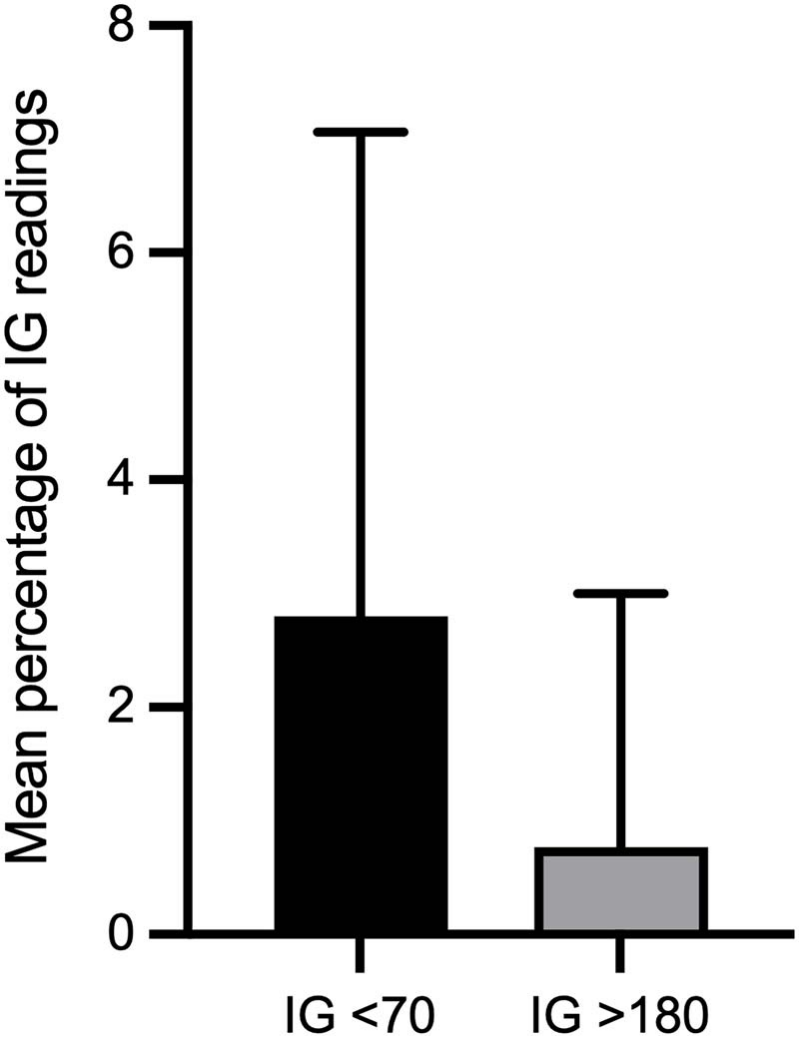
Mean ± SD percentage of IG concentrations per dog within the low IG (<70 mg/dL) and high IG (>180 mg/dL) ranges (*n* = 19 dogs). Abbreviation: IG = interstitial glucose.

**Figure 2:**
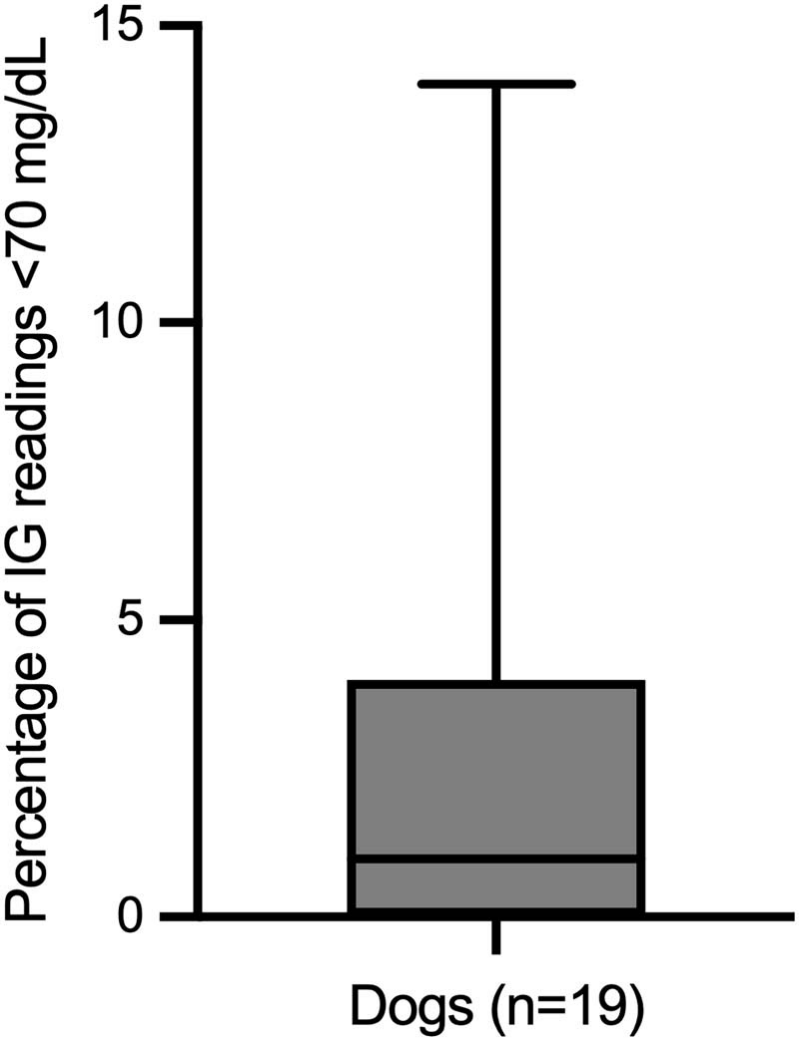
Distribution of percentage of IG concentrations classified as low IG (<70 mg/dL). The box represents the interquartile range, the horizontal line is the median, and whiskers indicate the full range. Abbreviation: IG = interstitial glucose.

**Figure 3:**
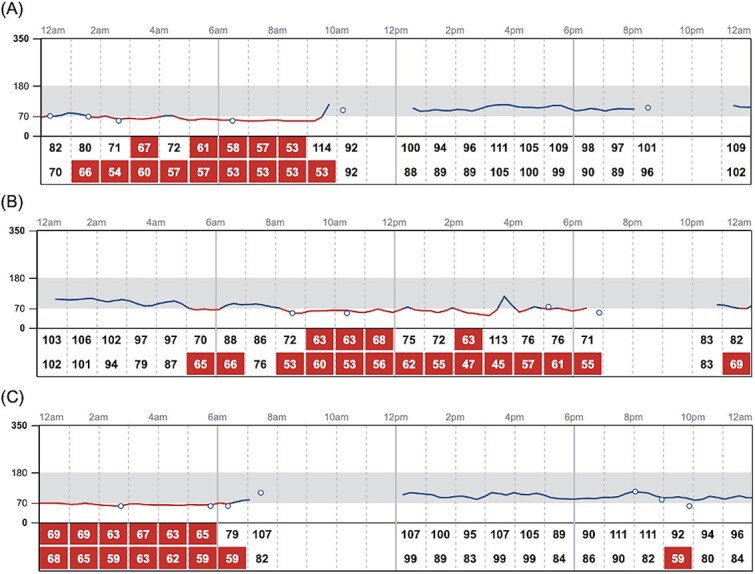
Representative IG curves from dogs with intermittent low IG concentrations. Red cells denote low IG values. Data were visualized using LibreView, the graphic interface for FreeStyle Libre devices. Dogs shown include (A) a 3-year-old, 2.9-kg female spayed Chihuahua, (B) a 4-year-old, 33.0-kg male neutered mixed breed, and (C) an 8-year-old, 5.0-kg male neutered Yorkshire Terrier. Abbreviation: IG = interstitial glucose.

**Figure 4:**
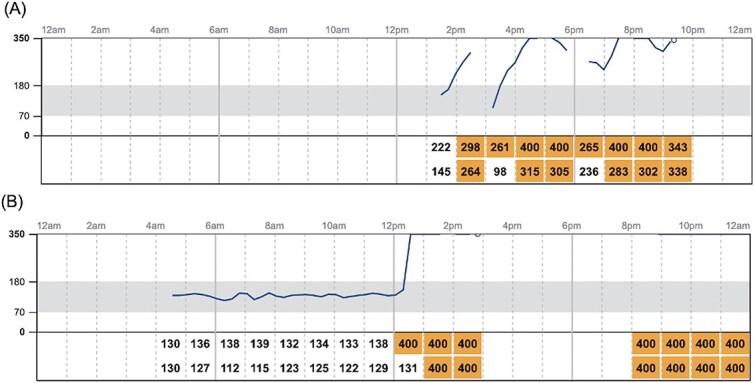
IG curves from dogs with markedly increased IG. Orange cells denote markedly increased IG values. Dogs shown include (A) a 5-year-old, 7.7-kg male neutered Cavalier King Charles Spaniel and (B) a 7-month-old, 29.0 kg male intact Doberman. Abbreviation: IG = interstitial glucose.

There were 16 180 IG samples analyzed. Across 19 healthy dogs, the pooled IG concentration distribution had a median of 107 mg/dL (IQR, 96-117 mg/dL; range, 40-400 mg/dL; mean, 107.8 mg/dL). The 2.5th and 97.5th percentiles of the pooled IG data were 69 mg/dL (90% CI, 68-69 mg/dL) and 143 mg/dL (90% CI, 142-144 mg/dL), respectively. During monitoring, 73.7% (14/19) of dogs experienced at least one low IG concentration (< 70 mg/dL), and the same proportion (14/19, 73.7) had at least one IG concentration above the upper descriptive percentile limit of 143 mg/dL. Using the FreeStyle Libre predefined cutoff, 26.3% (5/19) of dogs had at least one high IG concentration (>180 mg/dL). The mean (±SD) percentage of total low IG and high IG concentrations per individual was 2.8% ± 4.3% and 0.8% ± 2.2%, respectively (Figures 1 and 2). Markedly decreased IG concentrations (IG < 55 mg/dL) and markedly increased IG concentrations (IG > 250 mg/dL) were identified at least one time in 63.2% (12/19) and 10.5% (2/19) of dogs, respectively. Representative IG curves from dogs experiencing low IG and high IG are shown in Figures 3 and 4. A detailed summary of these events is provided in Table 1.

**Table 1:** Summary of IG events in 19 healthy dogs monitored with FGMS.

Glycemic category	Definition, mg/dL	Dogs with ≥1 event (%)	Mean % of readings per dog (±SD)
**Low IG**			
** Marked**	<55	63.2 (12/19)	0.6 ± 1.1
** Mild**	55-69	73.7 (14/19)	2.2 ± 3.3
** Normal**	70-180	100 (19/19)	96.4 ± 4.0
**High IG**			
** Mild**	181-250	26.3 (5/19)	0.2 ± 0.5
** Marked**	>250	10.5 (2/19)	0.6 ± 2.0

Abbreviations: FGMS = flash glucose monitoring systems; IG = interstitial glucose.

**Figure 5:**
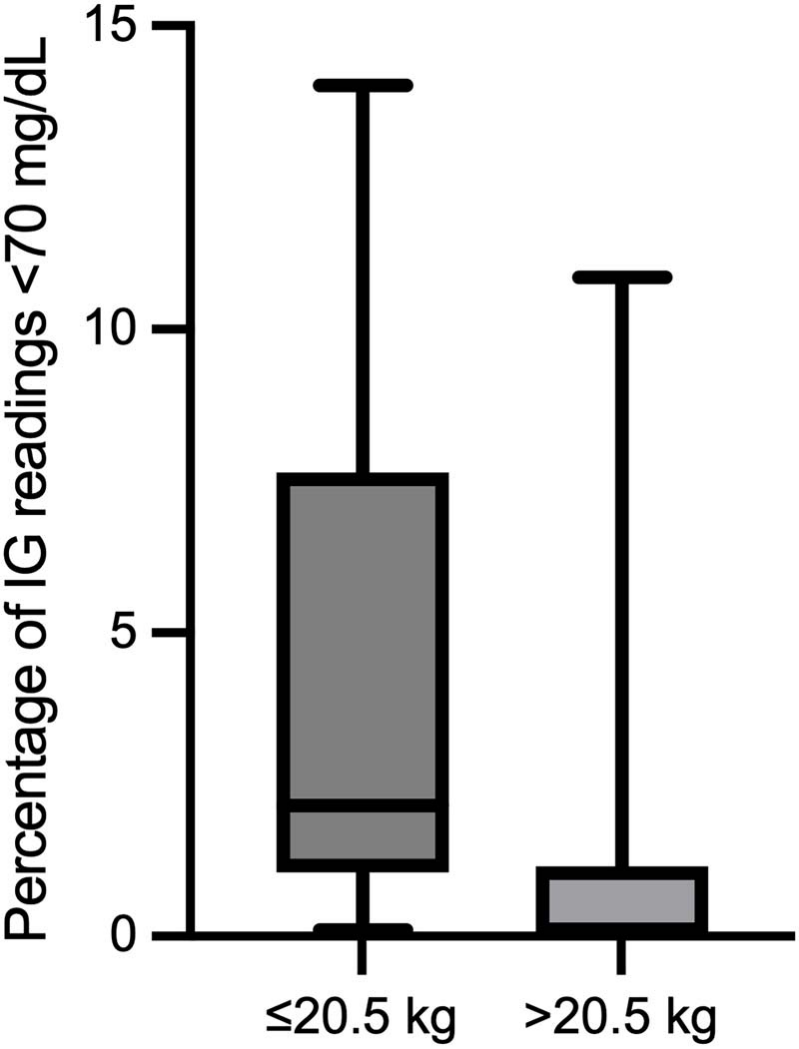
Percent of IG concentrations in the low IG range (<70 mg/dL) in dogs ≤20.5 kg (*n* = 9) and >20.5 kg (*n* = 10). Data are shown as box-and-whisker plots (median, IQR, and range). Smaller dogs experienced a significantly higher percentage of low IG concentrations than larger dogs (*P* = .02). Abbreviations: IG = interstitial glucose; IQR = interquartile range.

Demographic variables were analyzed to assess how low IG concentrations varied between groups. Dogs were divided into 2 weight cohorts: ≤20.5 kg (small dogs, *n =* 9) and >20.5 kg (large dogs, *n* = 10). The percentage of low IG concentrations differed significantly between groups (*P* = .02), with smaller dogs experiencing a higher median percentage of low IG concentrations (2.2%; IQR, 1.1-5.0) compared with larger dogs (0.1%; IQR, 0.0-0.7; Figure 5). The median difference was 2.1% (95% CI, 0.6-10.1).

When dividing dogs into age cohorts, 11 were ≤5 years old and 8 were >5 years old. The frequency of low IG concentrations between age groups (median difference, 0.9%; 95% CI, −8.2 to 3.5; *P =* .38) did not differ significantly.

Comparing dogs according to reproductive status (male intact, male neutered, and female spayed), no significant difference was found in the percentage of low IG concentrations (*P* = .82). Similarly, when grouped by sex (male vs female), the frequency of low IG concentrations did not differ significantly (median difference, 0.1%, 95% CI, −2.3 to 5.3; *P* = .71).

Two dogs experienced episodes of markedly increased IG concentrations (Figure 4) and underwent blood and urine testing within 10 days of these events. Both were euglycemic at the time of follow-up, had low-normal serum fructosamine concentrations, and had no evidence of glucosuria. According to the owners, the episodes occurred while the dogs were at home and were not associated with any known stressor or abnormal event. Neither dog exhibited clinical signs suggestive of hyperglycemia, such as polyuria, polydipsia, or polyphagia.

### Adverse events

All 23 sensor placements were performed without complication. No major adverse events were reported. Mild skin irritation or premature sensor detachment occurred in some dogs as previously described.^18^ Skin irritation was reported as mild and did not result in premature sensor removal in any dog. All enrolled dogs completed the study without requiring medical intervention related to the sensor.

## Discussion

We report the frequency of decreased and increased IG concentrations in healthy, nondiabetic dogs recorded using an FGMS. Of note, some healthy dogs experienced periods of naturally-occurring low and high IG concentrations, with low IG concentrations recorded more commonly. Because blood glucose concentrations were not measured concurrently, device accuracy could not be assessed. The frequency of low IG concentrations is consistent with expected physiologic variability in healthy individuals and does not necessarily indicate measurement error. These findings provide an important context for interpreting FGMS data in diabetic patients and emphasize that transient low or high IG concentrations may occur in healthy dogs without clinical abnormalities. Similar observations have been reported in healthy working dogs, where low IG concentrations occurred predominantly overnight without associated clinical signs, supporting that transient decreases in IG concentration may represent normal physiologic variation in dogs.^19^

The pooled IG concentration distribution in our study, characterized by descriptive percentile limits of 69-143 mg/dL, provides context for interpreting FGMS data in dogs. These findings further support the need for species-specific interpretation of FGMS results because human-derived glucose thresholds might not align with the IG concentration variation observed in healthy dogs. The similar proportions of dogs with IG concentrations below 70 mg/dL and above 143 mg/dL suggest that no single individual strongly skewed the percentile limits. However, because the sample size was small and each dog contributed many repeated measurements, some degree of disproportionate influence remains statistically possible despite outlier screening. In addition, because IG concentrations were collected under free-living conditions without standardized fasting periods, fasting-specific percentile limits could not be generated. Therefore, these percentile limits should be interpreted as descriptive summaries rather than transferable reference intervals.

Although the mean percentage of low IG concentrations per dog was only 2.8%, even a single low reading can affect management decisions for diabetic patients. When the FGMS detects a low IG concentration (<70 mg/dL), it triggers the default low glucose alarm, regardless of whether the pet is displaying clinical signs of hypoglycemia. Understandably, owners may become distressed and respond by feeding a small meal or by providing a rapidly absorbable carbohydrate source, either independently or under veterinary guidance. Additionally, these low IG concentrations can be perceived by the dog’s owner as a sign that diabetes mellitus is not adequately controlled, potentially prompting changes in insulin dosing. Therefore, even physiologic or transient low readings, whether or not they reflect true hypoglycemia, can meaningfully influence clinician and owner decision-making, emphasizing why awareness of these findings in healthy dogs is important when evaluating FGMS data in diabetic patients.

Unexpectedly, 2 dogs experienced markedly increased IG concentrations (IG > 250 mg/dL), despite being considered clinically healthy at the time of monitoring. In both cases, the events were transient and not associated with clinical signs. These excursions may have reflected physiologic postprandial fluctuations or sensor error rather than true pathologic hyperglycemia. Measurement variability has been reported in humans using the FreeStyle Libre system, with 12-15% of IG concentrations differing substantially from blood glucose concentration measurements, particularly within the first 24-48 hours of sensor wear.^20^ These observations emphasize the importance of contextual interpretation of FGMS data, particularly when unexpected results occur in the absence of clinical or biochemical abnormalities.

Our study also identified that smaller dogs experienced a significantly higher frequency of low IG concentrations compared with larger dogs. This observation could have been caused by a variety of factors. Individuals with a lower body mass index or less subcutaneous fat may experience discrepancies in IG measurements because of differences in sensor placement or interstitial fluid composition. Additionally, differences in sensor penetration depth relative to body size and fat distribution could influence IG measurements, further contributing to the observed differences between weight groups. Dogs with thinner skin may be particularly affected because decreased skin thickness has been shown to decrease FGMS accuracy and increase measurement bias, likely because of closer proximity of the sensor filament to underlying muscle and decreased interstitial fluid volume.^21^ Although the direction of this effect may differ, studies in humans similarly suggest that body composition influences CGMS performance, with higher body mass index associated with decreased device accuracy.^20,22–25^ Physiologic factors also may contribute to low IG concentrations independent of measurement bias. Smaller dogs generally have higher metabolic rates, lower glycogen reserves, and higher glucose turnover than larger dogs, making them inherently more susceptible to hypoglycemia.^23^ Collectively, these findings emphasize the importance of considering body weight when interpreting CGMS data in dogs. In contrast, age, reproductive status, and sex were not associated with the frequency of low IG concentrations in this cohort, suggesting that body weight was the only demographic factor evaluated that influenced IG variability. Future studies should explore whether breed-specific correction factors could improve the accuracy of CGMS readings across different body sizes. Additionally, inclusion of dogs with extreme BCSs (BCS 1-3 or 7-9) may help determine whether markedly low or high adiposity influences CGMS readings in dogs.

Our study had some limitations. First, there were gaps in IG data because of sensor dislodgement, sensor errors, and inconsistent owner compliance with scanning. These challenges represent real-world limitations with FGMS and may contribute to owner frustration. Additionally, the sample size was relatively small and limited to veterinary employee-owned dogs, which may not reflect the broader population that typically uses FGMS. Our study relied on owner-reported comorbidities and medications, which introduces the potential for incomplete or inaccurate background data. There was no cross-validation of FGMS readings with concurrent blood glucose concentration measurements. Therefore, there was no analysis of the accuracy of individual low or high readings. Finally, these results are specific to the FreeStyle Libre 2 and may not be generalizable to other devices.

In conclusion, although FGMS is widely used for the management of diabetes mellitus, we demonstrated that low and high IG concentrations can occur in healthy dogs. These findings emphasize the importance of cautious interpretation of IG concentration results, particularly in the absence of supportive clinical abnormalities. Integration of FGMS results with patient history, clinical signs, and conventional laboratory testing, along with thorough owner education, remains essential for appropriate clinical use.

## Supplementary Material

aalag032_Supplemental_Table_1_clean

## Off-label antimicrobial declaration

The authors declare no off-label use of antimicrobials.

## Institutional animal care and use committee or other approval declaration

Approved by the Internal Animal Care Committee of the Schwarzman Animal Medical Center, number AMC_12_5_24.

## Abbreviations

ALP: alkaline phosphatase
BCS: body condition score
CGMS: continuous glucose monitoring system
FGMS: flash glucose monitoring system
IG: interstitial glucose
