# Supplementary material for: Evaluation of low and high interstitial glucose concentrations in healthy, nondiabetic dogs using a flash glucose monitoring system
Source: J Vet Intern Med. 2026 Mar 6;40(2):aalag032. doi: 10.1093/jvimsj/aalag032 (PMC12963960; doi:10.1093/jvimsj/aalag032)
Supplement: aalag032_Supplemental_Table_1_clean [file aalag032_supplemental_table_1_clean.docx]

**Supplemental Table 1.** Breed, laboratory abnormalities, current medications, and comorbidities of study participants. All hematologic and biochemical abnormalities were classified as mild to moderate and were not considered likely to affect insulin sensitivity or glucose metabolism based on clinical interpretation. Some chemistry panels were collected post-prandially.

^†^Dog excluded from interstitial glucose analysis due to insufficient FGMS readings.

| Dog ID | Breed | Hematologic Abnormalities | Biochemical Abnormalities | Current Medications | Comorbidities |
| --- | --- | --- | --- | --- | --- |
| 1 | Cavalier King Charles Spaniel | None | Hypertriglyceridemia (210 mg/dL) | None | None |
| 2 | Mixed Breed | Anemia (36.4%) | Hypoproteinemia (5.4 g/dL), hypoalbuminemia (2.4 g/dL), hypercholesterolemia (357 mg/dL) | Fluoxetine | None |
| 3 | American Pit Bull Terrier | None | None | Trazodone | Myasthenia gravis (in remission) |
| 4^†^ | Beagle | None | Hypocalcemia (8.0 mg/dL) | None | None |
| 5^†^ | Golden Doodle | None | Hypochloremia (107 mmol/L), hypercholesterolemia (440 mg/dL) | None | None |
| 6 | Mixed Breed | None | Elevated ALP (679 U/L) | Joint supplement, Amandatidine, Cannabidiol (CBD) supplement | Osteoarthritis |
| 7 | Yorkshire Terrier | None | Hyperalbuminemia (4.1 g/dL), hypertriglyceridemia (880 mg/dL) | None | None |
| 8 | Mixed Breed | None | None | Fluoxetine | None |
| 9^†^ | Golden Doodle | None | None | None | Atopy, crystalluria |
| 10 | Border Collie | None | None | None | None |
| 11 | Mixed Breed | None | None | Probiotic | Grade I low grade mast cell tumor (completely excised) |
| 12 | Mixed Breed | None | Elevated AST (59 U/L) | None | None |
| 13 | Mixed Breed | Erythrocytosis (59.4%), reticulocytosis (126 K/µL) | Hyperglycemia (125 mg/dL) | None | None |
| 14 | Whippet | None | Hypoglycemia (58 mg/dL) | None | None |
| 15 | Yorkshire Terrier | None | Hyperglycemia (142 mg/dL) | None | None |
| 16 | Doberman | Eosinophilia (1.88 K/µL) | Hyperphosphatemia (7.7 mg/dL) | Trazodone | None |
| 17^†^ | Golden Retriever | None | Hypercholesterolemia (529 mg/dL), hypertriglyceridemia (239 mg/dL) | Cetirizine | None |
| 18 | Mixed Breed | Lymphocytosis (6.002 K/µL) | None | Probiotic | None |
| 19 | Standard Poodle | Lymphocytosis (5.85 K/µL) | None | Fluoxetine | None |
| 20 | Mixed Breed | None | None | None | None |
| 21 | Mixed Breed | None | None | None | None |
| 22 | Mixed Breed | Lymphopenia (0.516 K/µL) | None | None | None |
| 23 | Labrador Retriever | Neutropenia (2.776 K/µL) | None | Oclacitinib | Atopy |

Reference ranges for abnormal values (IDEXX, Westbrook, ME): hematocrit. 38.3*–*56.5%, reticulocytes 10*–*110 K/µL, neutrophils 2.94–12.67 K/µL, lymphocytes 1.06-4.95 K/µL, eosinophils 0.07–1.49 K/µL, triglycerides 20–150 mg/dL, cholesterol 131–345 mg/dL, glucose 63–113 mg/dL, phosphorus 2.5–5.1 mg/dL, calcium 8.4–11.8 mg/dL, chloride 108–119 mmol/L, total protein 5.5–7.5 g/dL, albumin 2.7–3.9 g/dL, AST 16–55 U/L, ALP 5–160 U/L.
